# Supplementary material for: Sex Differences in Mate Choice Preference Characteristics of Aequidens rivulatus
Source: Animals (Basel). 2022 May 7;12(9):1205. doi: 10.3390/ani12091205 (PMC9101118; doi:10.3390/ani12091205)
Supplement: Supplementary file 1 [file animals-12-01205-s001.zip › Supplementary Figure.pdf]

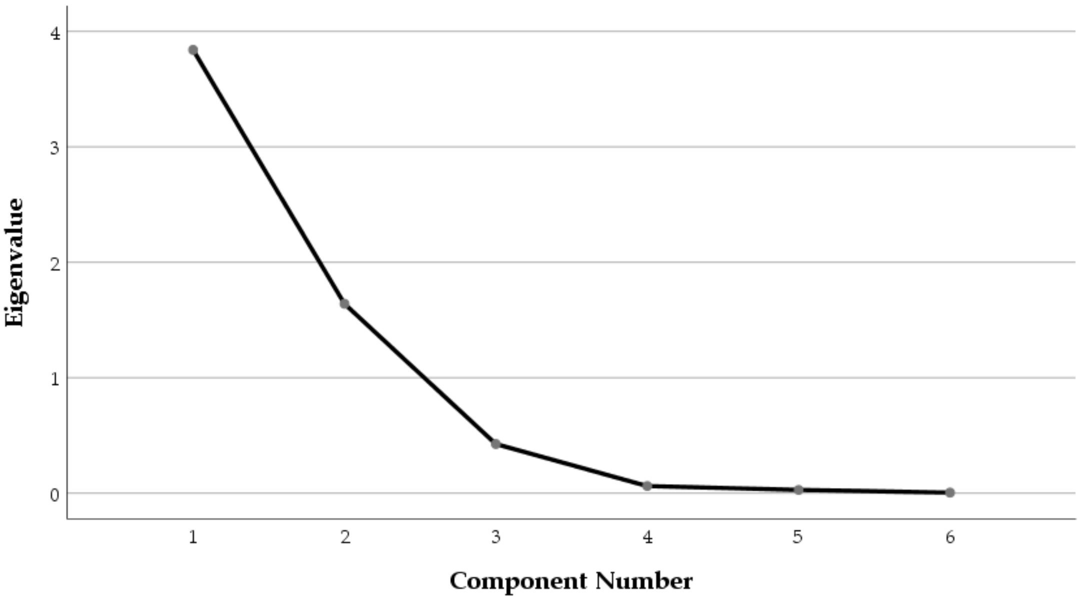

Figure S1: Scree plot of the preferred females of experiment 1.

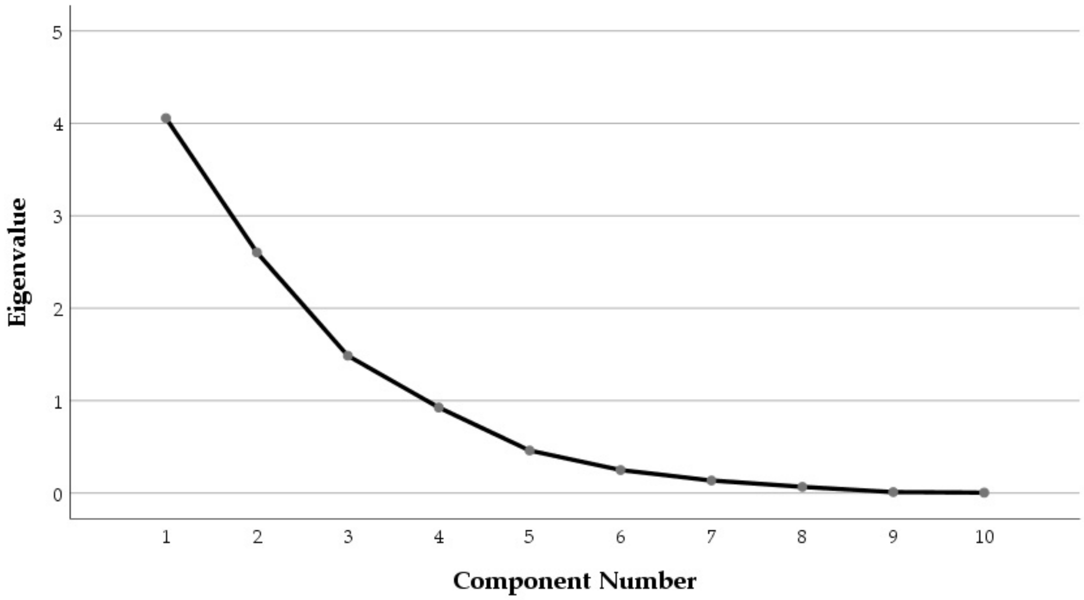

Figure S2: Scree plot of the main preference of males in experiment 1.

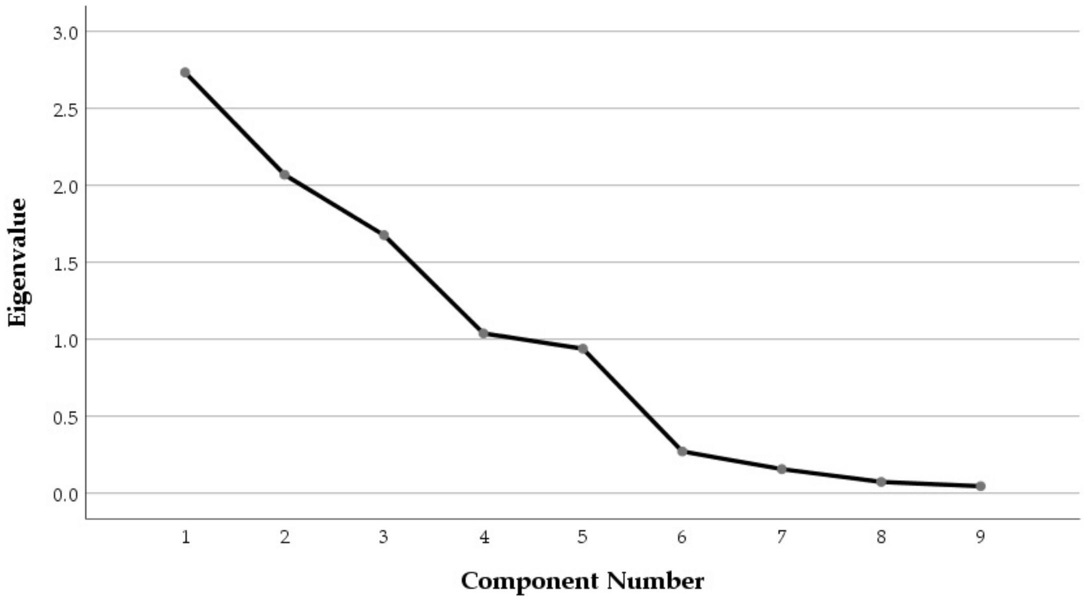

Figure S3: Scree plot of the preferred males of experiment 2.

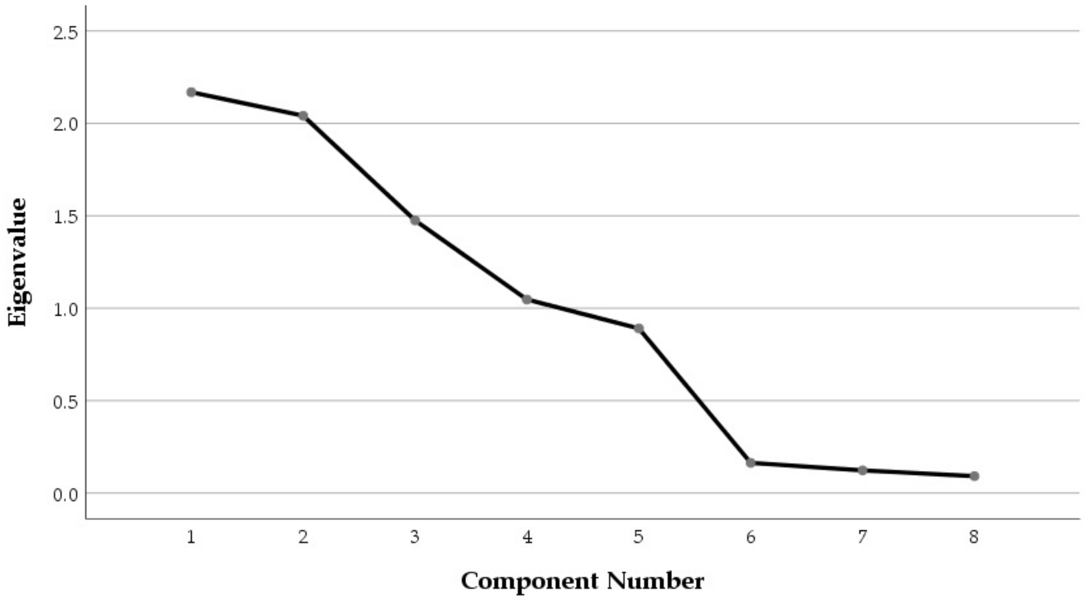

Figure S4: Scree plot of the non-preferred males of experiment 2.

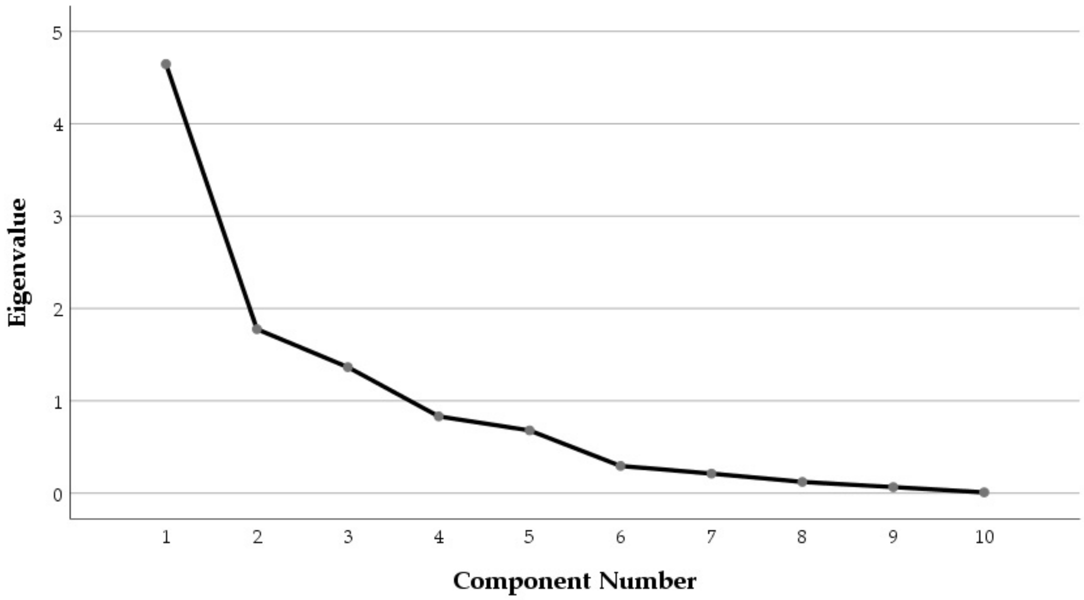

Figure S5: Scree plot of the main preference of females in experiment 2.
