# Supplementary material for: Sex Differences in Mate Choice Preference Characteristics of Aequidens rivulatus
Source: Animals (Basel). 2022 May 7;12(9):1205. doi: 10.3390/ani12091205 (PMC9101118; doi:10.3390/ani12091205)
Supplement: Supplementary file 1 [file animals-12-01205-s001.zip › Supplementary Table.pdf]

Table S1. KMO and Bartlett's test of the preferred females of experiment 1.

Kaiser-Meyer-Olkin Measure of Sampling Adequacy.		0.62
Bartlett's Test of Sphericity	Approx. Chi-Square	216.04
	df	15
	Sig.	0.00

Table S2. KMO and Bartlett's test of the main preference of males in experiment 1.

Kaiser-Meyer-Olkin Measure of Sampling Adequacy.		0.56
Bartlett's Test of Sphericity	Approx. Chi-Square	605.72
	df	45
	Sig.	0.00

Table S3. KMO and Bartlett's test of the preferred males of experiment 2.

Kaiser-Meyer-Olkin Measure of Sampling Adequacy.		0.55
Bartlett's Test of Sphericity	Approx. Chi-Square	143.49
	df	36
	Sig.	0.00

Table S4. KMO and Bartlett's test of the non-preferred males of experiment 2.

Kaiser-Meyer-Olkin Measure of Sampling Adequacy.		0.50
Bartlett's Test of Sphericity	Approx. Chi-Square	69.94
	df	21
	Sig.	0.00

Table S5. KMO and Bartlett's test of the main preference of females in experiment 2.

Kaiser-Meyer-Olkin Measure of Sampling Adequacy.		0.60
Bartlett's Test of Sphericity	Approx. Chi-Square	197.33
	df	45
	Sig.	0.00
